# Bilateral Paraventricular Nucleus Upregulation of Extracellular Superoxide Dismutase Decreases Blood Pressure by Regulation of the NLRP3 and Neurotransmitters in Salt-Induced Hypertensive Rats

**DOI:** 10.3389/fphar.2021.756671

**Published:** 2021-11-25

**Authors:** Qing Su, Xiao-Jing Yu, Xiao-Min Wang, Hong-Bao Li, Ying Li, Juan Bai, Jie Qi, Nianping Zhang, Kai-Li Liu, Yan Zhang, Guo-Qing Zhu, Yu-Ming Kang

**Affiliations:** ^1^ Department of Physiology and Pathophysiology, Xi’an Jiaotong University School of Basic Medical Sciences, Shaanxi Engineering and Research Center of Vaccine, Key Laboratory of Environment and Genes Related to Diseases of Education Ministry of China, Xi’an, China; ^2^ Department of Anesthesiology and Center for Brain Science, The First Affiliated Hospital of Xi’an Jiaotong University, Xi’an, China; ^3^ Department of Clinical Medicine, Medical School of Shanxi Datong University, Datong, China; ^4^ Key Laboratory of Targeted Intervention of Cardiovascular Disease, Collaborative Innovation Center of Translational Medicine for Cardiovascular Disease, Department of Physiology, Nanjing Medical University, Nanjing, China

**Keywords:** salt-induced hypertension, Ec-SOD, NLRP3, neurotransmitters, paraventricular nucleus

## Abstract

**Aims:** Long-term salt diet induces the oxidative stress in the paraventricular nucleus (PVN) and increases the blood pressure. Extracellular superoxide dismutase (Ec-SOD) is a unique antioxidant enzyme that exists in extracellular space and plays an essential role in scavenging excessive reactive oxygen species (ROS). However, the underlying mechanism of Ec-SOD in the PVN remains unclear.

**Methods:** Sprague–Dawley rats (150–200 g) were fed either a high salt diet (8% NaCl, HS) or normal salt diet (0.9% NaCl, NS) for 6 weeks. Each group of rats was administered with bilateral PVN microinjection of AAV-Ec-SOD (Ec-SOD overexpression) or AAV-Ctrl for the next 6 weeks.

**Results:** High salt intake not only increased mean arterial blood pressure (MAP) and the plasma noradrenaline (NE) but also elevated the NAD(P)H oxidase activity, the NAD(P)H oxidase components (NOX2 and NOX4) expression, and ROS production in the PVN. Meanwhile, the NOD-like receptor protein 3 (NLRP3)–dependent inflammatory proteins (ASC, pro-cas-1, IL-β, CXCR, CCL2) expression and the tyrosine hydroxylase (TH) expression in the PVN with high salt diet were higher, but the GSH level, Ec-SOD activity, GAD67 expression, and GABA level were lower than the NS group. Bilateral PVN microinjection of AAV-Ec-SOD decreased MAP and the plasma NE, reduced NAD(P)H oxidase activity, the NOX2 and NOX4 expression, and ROS production, attenuated NLRP3-dependent inflammatory expression and TH, but increased GSH level, Ec-SOD activity, GAD67 expression, and GABA level in the PVN compared with the high salt group.

**Conclusion:** Excessive salt intake not only activates oxidative stress but also induces the NLRP3-depensent inflammation and breaks the balance between inhibitory and excitability neurotransmitters in the PVN. Ec-SOD, as an essential anti-oxidative enzyme, eliminates the ROS in the PVN and decreases the blood pressure, probably through inhibiting the NLRP3-dependent inflammation and improving the excitatory neurotransmitter release in the PVN in the salt-induced hypertension.

## Introduction

Long-term excessive salt intake is one of the high-risk environmental factors responsible for the blood pressure regulation and the major dietary determinant for cardiovascular diseases especially the salt-induced hypertension. Numerous studies have reported that excessive amounts of sodium (NaCl) intake elevated the reactive oxygen species (ROS) production and restrained the antioxidant capacity at once, which would cause the target organ damages such as vascular endothelial cell, renal tubules, myocardial performance, and central nervous system (Nurkiewicz and Boegehold, 2007; Gu et al., 2008; Gu et al., 2009; Boegehold et al., 2015). As we know, high salt intake increases the delivery of sodium to renal tubules, activates the renin-angiotensin system (RAS), and promotes sodium and water re-absorption from renal tubules, which brings about oxidative stress and proinflammatory cytokines in endothelial cells and then develops to cardiorenal syndrome and hypertension (Weinstock et al., 1996; Selektor and Weber, 2008; Fujita et al., 2012). In addition, excessive sodium intake raises the endothelial NAD(P)H oxidase-dependent oxidative stress, resulting in the excessive amount of superoxide anion production that is mainly injurious to the vasculature (Matsui et al., 2006; Fujita et al., 2007; Kido et al., 2008). Also, all of those disturb the signaling pathways for endothelial nitric oxide synthase (NOS) activity so far as to make a reduction of the vascular NO bioavailability and protective function for the cardiovascular system (Channon, 2004; Boegehold, 2013). Therefore, WHO recommends that the suggested daily salt intake of the general population is 6–8 g, which is suitable for our body health. Patel and Joseph (2020) and other studies pointed out that high sodium intake can decrease α/β myosin heavy chain ratio by increasing the β expression, which causes the decrease of myocardial mechanical performance (Patel and Joseph, 2020). Meanwhile, high sodium diet has a negative effect on the Ca^2+^ and Na^+^ exchange, which is vital to main excitation–contraction coupling and cardiac mechanical performance. In the brain, excess dietary salt increases the concentration of sodium in the brain that leads to endogenous ouabain (EO) release while the EO inhibits the Na^+^/K^+^-ATP pump and increases angiotensin II (ANG II) in the central system, which significantly elevates the sympathetic excitation and the blood pressure (Blaustein et al., 2012). Therefore, high salt intake is closely associated with hypertension and cardiovascular diseases.

Numerous studies have reached an agreement that high salt diet leads to oxidative stress including the peripheral organs and central system (Huang and Leenen, 1996; 1999). In the brain, excessive sodium elevates the NAD(P)H-oxidase activity so as to generate excessive amounts of ROS. Our previous studies have indicated that high salt diet triggered oxidative stress in the paraventricular nucleus of hypothalamus (PVN) and increased the sympathetic activity, but blood pressure was decreased after bilateral paraventricular nucleus infusion of oxygen radical scavenger (temple), which means oxidative stress in the PVN contributes to sympathetic excitation and blood pressure (Su et al., 2014). Superoxide dismutase (SOD) is a broadly known essential antioxidant enzyme, which can catalyze two superoxide ions and two hydrogen ions to form one oxygen molecule and one hydrogen peroxide molecule. The generated hydrogen peroxide is further decomposed into water molecule and oxygen molecule under catalase (CAT) catalysis. There are three members of the SOD family including cytosolic SOD (Cu/Zn-SOD, SOD1), mitochondrial SOD (Mn-SOD, SOD2), and extracellular SOD (Ec-SOD, SOD3). As for the Ec-SOD, it plays a primary anti-oxidative defense for the oxidative stress and takes part in the cellular signal transduction, and it is also a unique antioxidant enzyme that exists in extracellular space (Marklund et al., 1982; Sasaki et al., 2021). However, there are no abundant evidences for this newcomer antioxidant enzyme. Therefore, we intend to explore the mechanism of Ec-SOD on excessive salt intake–induced hypertension.

As for the inflammation, there are numerous evidences showing that oxidative stress induced the inflammation in the PVN (Su et al., 2016) and inflammasome is mainly a step to act on the chronic inflammation responses. Schroder and Tschopp (2010) showed that NOD-like receptor protein 3 (NLRP3) inflammasome is a molecular platform activated upon signs of cellular “danger” to trigger innate immune defenses through the maturation of pro-inflammatory cytokines such as interleukin (IL)-1β (Schroder and Tschopp, 2010; Jo et al., 2016). NLRP3 inflammasome-mediated inflammation takes part in neurologic diseases and can be activated by damaged mitochondria releasing ROS (Zhou et al., 2011; Alsaadi et al., 2021). Therefore, we wonder whether the ROS in the PVN activated the NLRP3 inflammasome-mediated inflammation and finally induced the inflammatory cytokine expression in the PVN.

In addition, paraventricular nucleus of the hypothalamus exists in the presympathetic neurons and is a primitive action for modulating the downstream sympathetic nerve activity, which is tightly linked to blood pressure (Thorsdottir et al., 2021). Numerous evidences demonstrated that salt diet elevated the noradrenaline (NE) level and tyrosine hydroxylase (TH) expression in the PVN during the process of hypertension (Wang et al., 2018; Yu et al., 2019). TH, which can catalyze the conversion of l-tyrosine to l-3,4-dihydroxyphenylalanine (L-DOPA), is the initial and rate-limiting step in the biosynthetic pathway of catecholamines including dopamine, NE, and adrenaline. NE is released predominantly from the ends of sympathetic nerve fibers and acts to increase the force of skeletal muscle contraction and the rate and force of contraction of the heart so as to increase the blood pressure. Therefore, the high level of TH can increase catecholamine production, especially NE, which elevates the sympathetic activity and blood pressure. As we know, glutamic acid decarboxylase (GAD) is the crucial enzyme involved in the synthesis of gamma-aminobutyric acid (GABA), a major inhibitory neurotransmitter of the central nervous system. Therefore, once the neurons were stimulated by acute or chronic factors like ROS, the resting potential of neurons were triggered to conduct the action potentials and transmits the neurotransmitters between prominences increasing the excitatory (including NE and epinephrine, Glu) release and suppressing the inhibitory neurotransmitter secretion such as GABA. Also, those excitatory neurotransmitters are released predominantly from the ends of sympathetic nerve fibers and then augmented the sympathetic nerve activity. All of those made us to explore whether the ROS in the PVN can regulate the neurotransmitters and finally decrease the sympathetic nerve activity and blood pressure in salt-induced hypertension.

In summary, high salt diet induced the oxidative stress in the PVN, but how Ec-SOD regulates the sympathetic nerve activity and blood pressure remains unclear. Some researchers have indicated that ROS is closely related to inflammatory cytokines and neurotransmitters and probably takes part in the development of hypertension. Therefore, the aim of this study is to determine whether the overexpression of Ec-SOD in the PVN decreases the blood pressure *via* regulation of the NLRP3 pathway and neurotransmitters in salt-induced hypertension.

## Materials and Methods

### Animals

Sprague–Dawley rats weighing 150–200 g were purchased from the experimental animal center of Xi’an Jiaotong University. All animals were housed in temperature-controlled (23 ± 2°C) and light-controlled (12 h light/dark cycle) animal quarters and were provided with rat chow *ad libitum*. The study was approved by the Animal Care and Use Committee of Xi’an Jiaotong University (No. 2018-404). The design conformed to the Guide for the Care and Use of Laboratory Animals published by the United States National Institutes of Health (the US National Institutes of Health Publication No. 85-23, revised 1996).

### Adenovirus-Associated Virus Preparation

The Ec-SOD plasmid vector (HBAAV2/9-CMV-MCS-Zsgreen) containing the gene of *Rattus norvegicus* Ec-SOD targeting sequence (Gene ID: 25352; Transcript: NM_012880.2, 731 bp) and green fluorescent protein was provided by Hanbio Biotechnology Co. Ltd (Shanghai, China, contract number: HH20210317ZY-AAV01). The related information is in Supplementary Figure S1. The titer of the adenovirus-associated virus (AAV) is 1 × 10^12^ μg/ml and the serotype is AAV 9. After we obtained the adenovirus-associated virus, the AAV would be subpackaged (200 μl/tube) and store at −80°C. Before bilateral paraventricular nucleus injection of AAV-Ec-SOD or AAV-Ctrl, the vectors should be dissolved on ice.

### Surgery and Stereotaxic Injection

For vector injection, rats were anesthetized with a ketamine (80 mg/kg) and xylazine (10 mg/kg) mixture (intraperitoneally), and the head was placed into a stereotaxic apparatus. The microinjector (5 μl) with vectors (either AAV-Ec-SOD or AAV-Ctrl) was installed in the microinjection pump. Vectors were bilaterally injected into the PVN (coordinates: ±0.4 mm from midline, −1.8 mm posterior to bregma, and −7.9 mm ventral to dura according to the Paxinos and Watson rat brain atlas) using 2 μl volume each side at the rate of 0.2 μl/min (He et al., 2017; Zhu et al., 2018; Zheng et al., 2020). After finishing injection, the needle was kept in the site for 10 min and then moved away. Then the holes were covered with dental tray powder. Also, the wounds were treated with antibiotics and finally stitched. After surgery, animals recovered from the anesthetic and cefotaxime sodium (80 mg/kg) was applied through intraperitoneal injection twice a day in the following 7 days (Bai et al., 2017a; Huo et al., 2020).

### General Experimental Protocol

**FIGURE 1:**
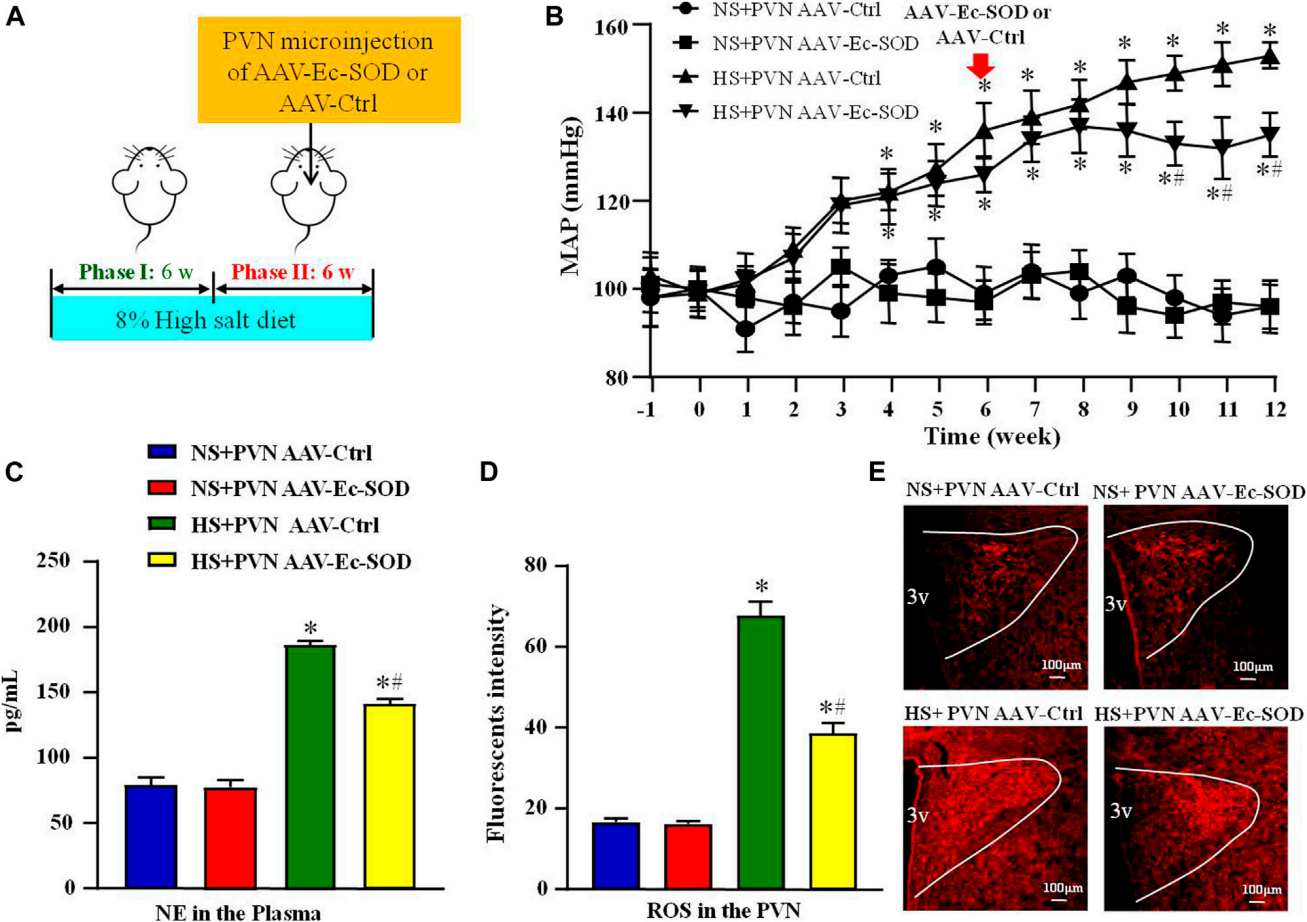
Effect of AAV-Ec-SOD on mean arterial blood pressure, plasma noradrenaline and PVN level of superoxide anion **(A)** Schematic diagram for the experimental protocol; **(B)** Line chart comparing the effect of AAV-Ec-SOD on mean arterial blood pressure (MAP) during the 12 weeks; **(C)** Column diagram analysis of the plasma noradrenaline (NE) in different groups; **(D–E)** Immunofluorescence for dihydroethidium (DHE) and Column diagram showing the immunofluorescent intensity of DHE in the PVN in different groups. 3V represents the third ventricle in brain. Values are expressed as mean ± SEM (n = 6). *p < 0.05 *vs.* NS groups (NS + PVN AAV-Ctrl or NS + AAV-Ec-SOD); ^***#***^ *p* < 0.05 *vs*. HS + PVN AAV-Ctrl.

Sprague–Dawley rats (150–200 g) were fed with 8% high salt diet (8% NaCl, HS) and 0.9% normal salt diet (0.9%, NaCl, NS) for 6 weeks. Then those two groups were respectively separated into AAV-Ec-SOD and AAV-Ctrl (empty vector) groups. Each group of rats was administered with bilateral PVN microinjection of the overexpression of AAV-Ec-SOD or AAV-Ctrl once at the beginning of the seventh week. High salt diet or normal salt diet was continued for a total of 12 weeks (Figure 1A) and the available number of each group rat is 6. All groups are as follows: normal salt + PVN AAV-Ctrl (NS + PVN AAV-Ctrl); normal salt + PVN AAV-Ec-SOD (NS + PVN AAV-Ec-SOD); high salt + PVN AAV-Ctrl (HS + PVN AAV-Ctrl); high salt + PVN AAV-Ec-SOD (OH + PVN AAV-Ec-SOD). At the end of the study, after rats were anesthetized, blood was collected from the abdominal aortic vein and centrifuged at 3,000 r/min to obtain the plasma. One part of fresh brain tissues was put into 4% paraformaldehyde for 3 days and then moved into 30% sucrose for dehydration. The plasma, OTC embedding brains, and the rest of the fresh brain were stored at −80°C for the next molecular experiments.

### Blood Pressure Measurement

The method for the measurement of blood pressure was noninvasive *via* tail-cuff instrument, and their recording system has been described previously (Elks et al., 2011; Li et al., 2020; Xia et al., 2021). All animals were habituated to the blood pressure system and to the holders daily for 1 week prior to the initiation of experimental measurements. Unanesthetized rats were warmed to an ambient temperature of 30°C by placing them in a holding device mounted on a thermostatically controlled warming plate. Rat tail was allowed to accommodate the cuff for 10 min before blood pressure measurement. After that, the tail was threaded through the VPR sensor cuff and placed within 2 cm of the occlusion cuff. The temperature of the animals should be between 32 and 35°C. When the software displays a stable jagged wave, blood pressure could be measured. Appropriate training for software operation and animal handling is required prior to performing this method for BP measurement. The rats’ arterial pressure was measured every week during 12 weeks. Mean arterial pressure data were recorded 20 times, which were collected for 30 min between the same time (8:00 and 11:00), and those data were then averaged until the end of this study.

### Immunofluorescence and Immunohistochemistry

Immunofluorescence and immunofluorescence double staining were conducted according to the antibody instruction and it has been described previously (Xie et al., 2013). OTC embedding brains were sectioned into several 14-μm transverse sections from bregma −0.92 to −2.12 mm at −25°C by freezing microtome (Leica, CM1860). Sections were then washed in PBS containing 0.1% Tween 20 three times for 5 min, permeabilized in 0.3% Triton in Tris-buffered saline for 1 h at 37°C, blocked using 5% goat serum with 0.2% Triton in Tris-buffered saline for 1 h, and incubated with primary antibody in blocking buffer at 4°C overnight. The following primary antibodies were used in this section: NLRP3 (1:300, ab214185; Abcam), NOX2 (dilution 1:200, ab280952; Abcam), TH (1:400, ab6211; Abcam), and DAPI (1:500, ab254259; Abcam). After washing three times for 5 min, the sections were incubated with secondary antibodies (Alexa Fluor-conjugated donkey anti-mouse or anti-rabbit IgG (1:200; Molecular Probes)), washed in PBS three times for 5 min, and incubated in DAPI (4′,6-diamidino-2-phenylindole, Invitrogen) for 30 min. For double-label immunofluorescence staining, sections were stained with the two primary antibodies listed previously and two different sources of secondary antibodies were also added. After adding the antifade solution, immunofluorescence staining sections were imaged using a fluorescence microscope (Nikon Eclipse, 80i, Japan) and double staining sections were observed on a laser scanning confocal microscope (Nikon C2, Japan).

For diaminobenzidine (DAB) staining, sections stained with primary antibody c-fos (1:300, ab222699; Abcam) were washed in PBS containing 0.1% Tween 20 for 1 h, then incubated with anti-rabbit IgG (HRP) secondary antibody (1:200, AB7090; Abcam) in blocking buffer for 1 h. The horseradish peroxidase reaction was detected using 3,3-diaminobenzidine (DAB) kit (P0203, Beyotime, China) according to the manufacturer’s instructions. Processing was stopped with H_2_O and sections were imaged using a microscope (Nikon Eclipse, 80i, Japan).

### Measurement of Superoxide Anion in PVN

The level of superoxide anion in the PVN was detected by dihydroethidium (DHE; Molecular Probes). Brain sections (frozen section, 14 μm) were incubated for 10 min with dihydroethidium (1 μmol/L; Sigma) at 37°C in the dark, as previously described. The oxidative fluorescence intensity was detected at 585 nm wavelength by fluorescence microscope imaging system (Nikon).

### Western Blotting

As for the western blotting procedures, many references have been described (Lupia et al., 2010). Microdissection procedures were used to isolate the PVN tissue. The tissues were collected from both sides of the PVN of individual rats. Protein extracted from PVN tissues were prepared as described previously (Lupia et al., 2010). Western blotting was used for measurement of protein expression. The tissues were then homogenized in 100 μl of RIPA lysis buffer containing protease inhibitor cocktail. The protein was extracted from the homogenates, and the protein concentration in the lysate was measured using a BCA assay. Protein extracts (30 μg) were combined with an equal volume of loading buffer, boiled for 5 min, and electrophoresed on 10–15% SDS-polyacrylamide gels. The proteins were electroblotted onto polyvinylidene PVDF membranes. Non-specific binding was blocked by incubating the membranes in 1% casein in PBS–Tween for 1 h at room temperature. Blots were then incubated overnight at 4°C with the primary antibodies. The primary antibodies used in this study are as follows: Ec-SOD (1:400, sc-271170; Santa Cruz), NOX2 (1:300, ab129068; Abcam), NOX4 (1:400, ab133303; Abcam), NLRP3 (1:300, ab263899; Abcam), ASC (1:300, ab180799; Abcam), VCAM-1 (1:400, ab115135; Abcam), pro-casp-1 (1:500, ab238972; Abcam), CXCR3 (1:200, ab71864; Abcam), IL-1β (1:400, ab283818; Abcam), CCL2 (1:300, ab186421; Abcam), TH (1:300, ab75875; Abcam), and GAD67 (1:200, ab213508; Abcam). After washing with wash buffer four times for 10 min each time, blots were then incubated for 1 h with secondary antibody (1:10,000 dilution; Santa Cruz Biotechnology) labeled with horseradish peroxidase. Immunoreactive bands were visualized using enhanced chemiluminescence (ECL Plus; Amersham). The β-actin antibody was used as an internal standard. Band densities were analyzed using ImageJ software.

### Quantitative Real-Time PCR Assays

Analysis of relative change in gene transcripts was performed by qPCR assay. The hypothalamic tissues were dissected as described previously. Briefly, rat brains were isolated and cut into a coronal segment (−0.92 to −2.13 mm posterior to bregma). A block of the hypothalamus containing PVN was excised from the coronal section. Total RNA was extracted from microdissected PVN using Tri-Zol reagent (Invitrogen, product #15596026) and reverse transcribed using oligo (dT) with conditions at 23°C for 10 min, 37°C for 60 min, and 95°C for 5 min. The cDNA was used for real-time PCR with specific primers for Ec-SOD (forward primer: 5′-ATGGTGGCCTTCTTG TTCTGC-3′, reverse primer: 5′-GTGCTGTGG GTG CGG CACACC-3′) and GAPDH (forward primer: 5′-AGA​CAG​CCG​CAT​CTT​CTT​GT-3′, reverse primer: 5′-CTT​GCC​GTG​GGT​AGA​GTC​AT-3′). The quantitative fold changes in mRNA expression were determined relative to glyceraldehyde-phosphate dehydrogenase (GAPDH) mRNA levels in each corresponding group.

### Enzyme-Linked Immunosorbent Assay

We used ELISA kits to measure plasma level of noradrenaline (NE, Abnova, KA3768) (Li et al., 2016; Bai et al., 2017b; McKie et al., 2019). Oxidative stress relevant indicators, including superoxide dismutase activity (Ec-SOD, ab277415; Abcam), NAD(P)H oxidase (ab186031; Abcam), malondialdehyde (MDA, S0131; Beyotime, China), and glutathione (GSH, A006-2-1; Nanjing Jiancheng Bioengineering, China) in PVN, were also measured with ELISA kits. The method has been described previously (Alsaadi et al., 2021).

### High-Performance Liquid Chromatography

We used the HPLC-EC system to measure the level of GABA in the PVN, and the specific procedures had been described previously (MohanKumar et al., 1998). Briefly, it consists of an UltiMate 3000 RS chromatograph (Thermo Fisher Scientific), Thermo TSQ Quantum Access MAX mass spectrometer (Thermo Fisher Scientific), a glassy carbon working electrode, and a 3-μm ODS reverse phase 150 × 4.6 mm C-18 column (Welch Ultimate XB-C18). The aqueous phase is 0.1% ethanoic acid/10 mM ammonium acetate and organic phase is acetonitrile. Automatic sampler volume was 1.0 μl and the sampler temperature stayed at 10°C. Also, the washing needle volume needs 200 μl methanol with 3.00 ms elapsed soak. The flow rate of the pump was 0.8 ml/min. The column and the working electrode were kept at a temperature of 40°C. Each reserve solution was diluted with 0.2% formic acid/methanol to the concentration ladder standards (GABA standards: 5,000, 1000, 100, 10, 5 ng/ml) and to get the standard curve. At the time of HPLC-EC analysis, 1 ml 0.2% formic acid/methanol was added to each sample and grinded for 3 min. After that, they were homogenized and centrifuged at 12,000 rpm for 10 min. Then 800 μl of supernatant was bent-dried with nitrogen, and the residue was re-dissolved with 100 μl 0.2% formic acid/methanol, centrifuged at 1200 rpm for 10 min, and finally 10 μl of supernatant was taken for sample analysis.

### Statistical Analysis

Data are presented as the mean ± SEM. Statistical analyses were performed using Prism version 8.0. MAP was analyzed by repeated measures ANOVA. One-way ANOVA followed by Tukey’s *post hoc* test was used to determine statistical differences in the number of positive neurons, fluorescent intensities, western blotting data, HPLC-EC, and ELISA tests.

## Results

### Effect of AAV-Ec-SOD on Mean Arterial Blood Pressure and Plasma Noradrenaline

To investigate the effect of Ec-SOD in the PVN on mean arterial blood pressure (MAP) and sympathetic activity, we measured the MAP *via* tail-cuff instrument and plasma noradrenaline (NE) by ELISA. The results showed that excessive salt intake increased MAP from the second week. Until the sixth week, the MAP was higher in salt-induced group than in normal salt group (99.83 ± 6.42 mmHg *vs*. 136.51 ± 5.67 mmHg, *p* < 0.05). Overexpression of Ec-SOD in the PVN significantly decreased the MAP of salt diet group at the end of the 12th week (153.17 ± 4.74 mmHg *vs*. 135.68 ± 5.10 mmHg, *p* < 0.05) (Figure 1B). As for the sympathetic activity indicator NE, high salt intake increased the level of NE in the plasma compared with the normal salt group (*p* < 0.05). Overexpression of Ec-SOD in the PVN decreased the plasma NE level in hypertensive group (*p* < 0.05) (Figure 1C).

### Effect of AAV-Ec-SOD on Oxidative Stress-Related Indicators in the PVN

To explore the effect of AAV-Ec-SOD on oxidative stress, we measured the PVN level of superoxide anion by DHE among the four groups. Excessive salt intake increased the level of superoxide anion in the PVN than the normal salt group (*p* < 0.05). After bilateral PVN microinjection of AAV-Ec-SOD, the PVN level of superoxide anion was lower than the high salt intake group (*p* < 0.05) (Figures 1D,E).

**FIGURE 2:**
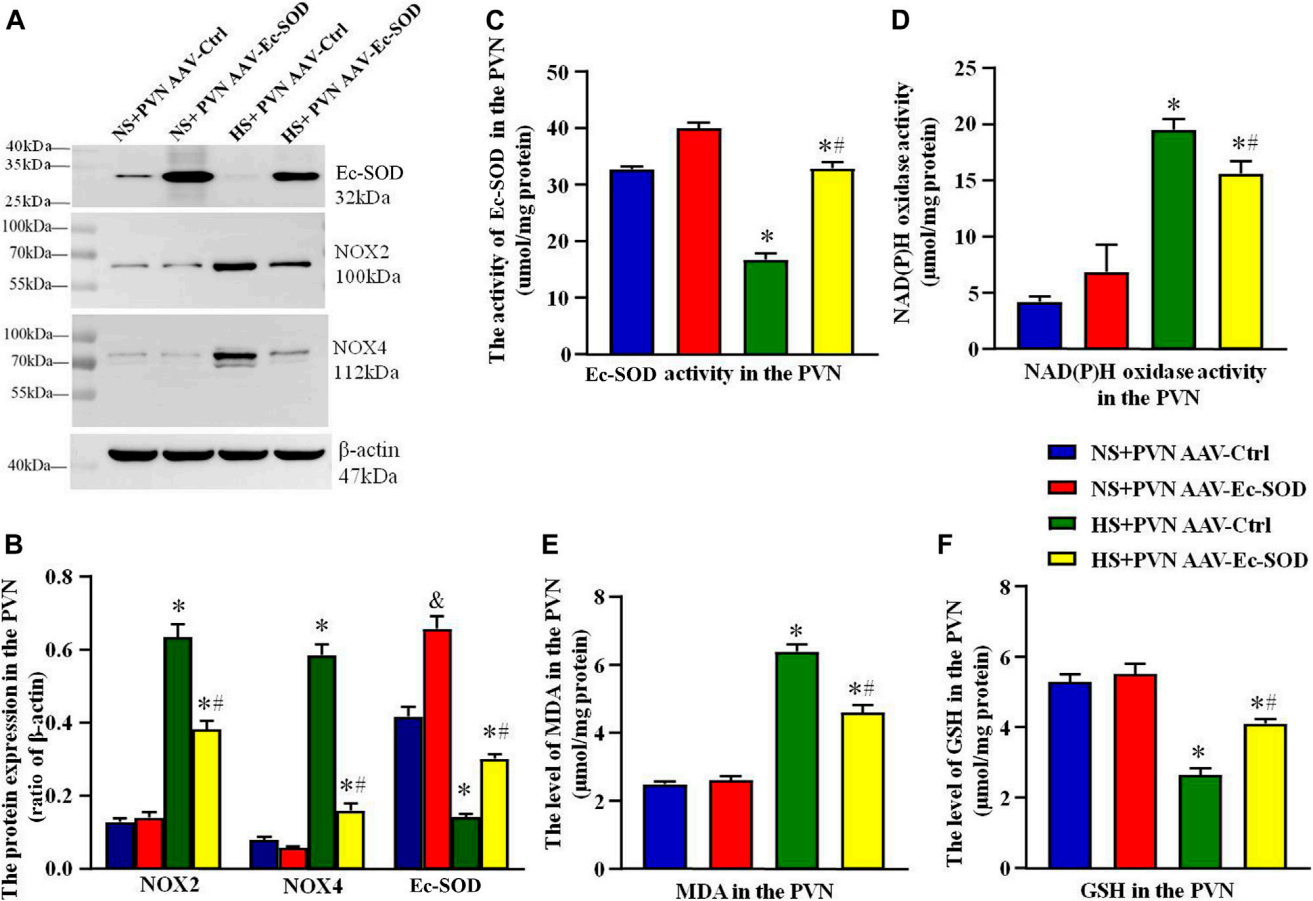
Effect of AAV-Ec-SOD on oxidative stress–related indicators in the PVN. **(A)** Representative immunoblot and **(B)** densitometric analysis of PVN expression levels of Ec-SOD, NOX2, and NOX4 in different groups. **(C)** Column diagrams for Ec-SOD activity, **(D)** NAD(P)H oxidase activity, **(E)** MAD level, **(F)** GSH level in the PVN of different groups. Values are expressed as mean ± SEM (n = 4–6). ^&^ *p* < 0.05 AAV-Ctrl *vs.* AAV-Ec-SOD; *p < 0.05 NS + PVN AAV-Ctrl *vs.* HS + PVN AAV-Ec-SOD; ^***#***^ *p <* 0.05 HS + PVN AAV-Ctrl *vs.* HS + PVN AAV-Ec-SOD.

Western blotting results showed that the PVN expressions of NOX2 and NOX4 were higher in the excessive salt intake group than in the normal salt group (*p* < 0.05). After bilateral PVN microinjection of AAV-Ec-SOD, overexpression of Ec-SOD significantly decreased the PVN expression of NOX2 and NOX4 in the salt-induced hypertensive group (*p* < 0.05). As for the Ec-SOD expression in the PVN, bilateral PVN microinjection of AAV-Ec-SOD is significantly higher than AAV-Ctrl (*p* < 0.05) (Figures 2A,B).

We also measured the oxidative stress–related indicators including NAD(P)H oxidase activity, MDA level, and GSH level in the PVN by ELISA. Results indicated that NAD(P)H oxidase activity and MDA level in the PVN were higher in the excessive salt intake group than in the normal salt group (*p* < 0.05). Overexpression of Ec-SOD significantly decreased the NAD(P)H oxidase activity and MDA in the salt-induced hypertensive group (*p* < 0.05). As for the GSH level in the PVN, bilateral PVN microinjection of AAV-Ec-SOD in the salt-induced hypertensive group increased the GSH level compared with AAV-Ctrl (*p* < 0.05) (Figures 2D–F). In addition, the PVN of Ec-SOD activity in AAV-Ec-SOD group is higher than the AAV-Ec-SOD group. However, there is no significant change between the normal salt groups (*p* > 0.05) (Figure 2C).

### Effect of AAV-Ec-SOD on NLRP3 and c-fos-Positive Neurons in the PVN

**FIGURE 3:**
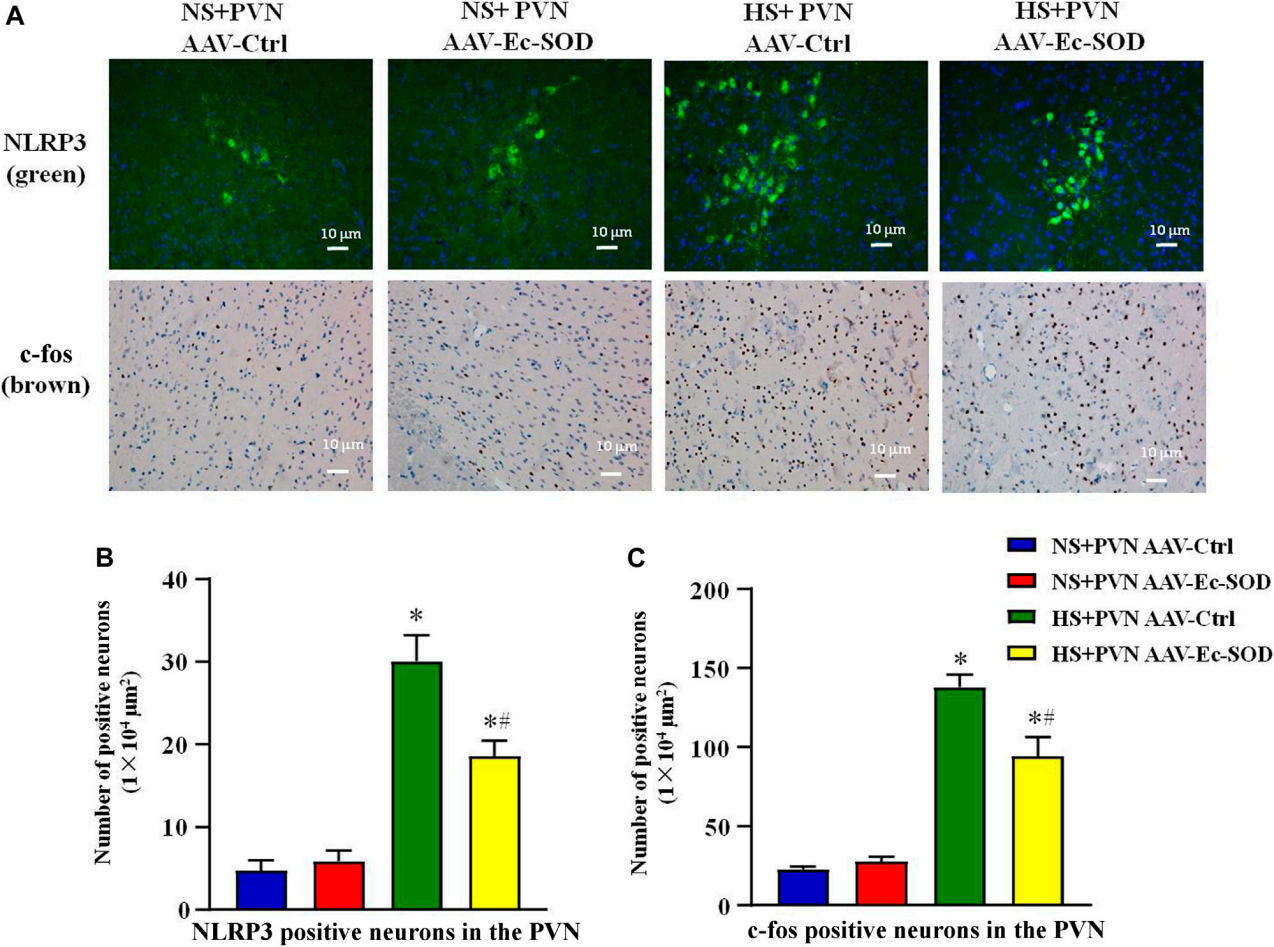
Effect of AAV-Ec-SOD on NLRP3 and c-fos-positive neurons in the PVN. **(A)** Immunofluorescence for NLRP3 and immunohistochemistry for c-fos positive neurons in the PVN in different groups. **(B–C)** Column diagram showing the NLRP3 and c-fos on positive neurons in the PVN in different groups. Values are expressed as mean ± SEM (n = 6). *p < 0.05 NS + PVN AAV-Ctrl *vs.* HS + PVN AAV-Ec-SOD; ^***#***^ *p <* 0.05 HS + PVN AAV-Ctrl *vs.* HS + PVN AAV-Ec-SOD.

We measured the PVN-positive neurons of NLRP3 and c-fos by fluorescence and immunohistochemistry staining. Excessive salt diet intake enhanced the number of NLRP3-positive neurons and c-fos-positive neurons in the PVN. However, bilateral PVN microinjection of AAV-Ec-SOD decreased the number of NLRP3-positive neurons and c-fos-positive neurons in the PVN of the salt-induced hypertensive group (*p* < 0.05) (Figures 3A–C).

### Effect of AAV-Ec-SOD on NOX2 and ASC-Positive neurons in the PVN

**FIGURE 4:**
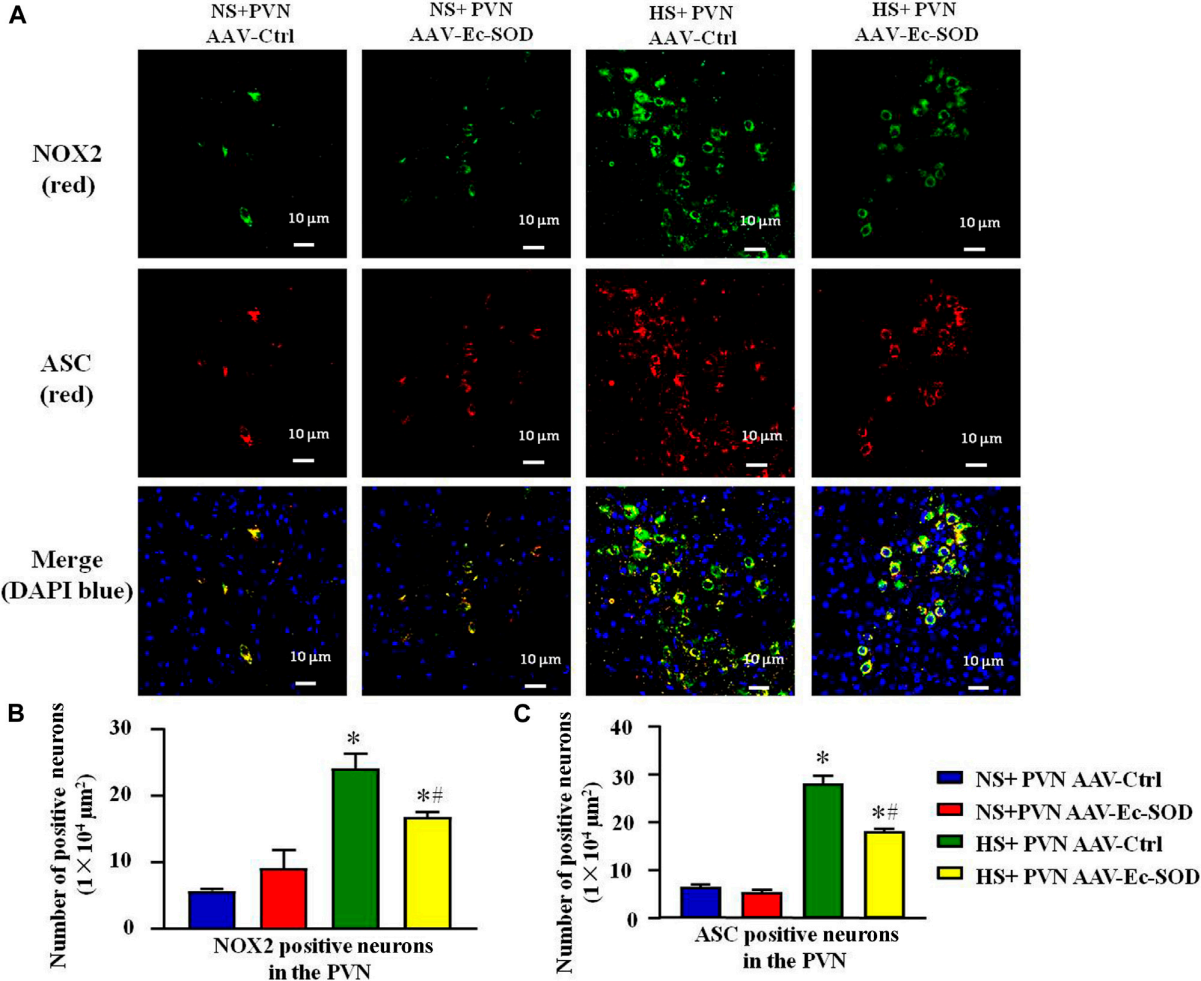
Effect of AAV-Ec-SOD on NOX2 and ASC-positive neurons in the PVN. **(A)** Double fluorescence staining for NOX2- and ASC-positive neurons in the PVN in different groups. **(B–C)** Column diagram showing the NOX2 and ASC-positive neurons in the PVN in different groups. Values are expressed as mean ± SEM (n = 6). *p < 0.05 NS + PVN AAV-Ctrl *vs.* HS + PVN AAV-Ec-SOD; ^***#***^ *p <* 0.05 HS + PVN AAV-Ctrl *vs.* HS + PVN AAV-Ec-SOD.

According to the aforementioned results, we measured PVN-positive neurons of NOX2 and ASC by double fluorescence staining. Excessive salt intake enhanced the number of NOX2-positive neurons and ASC-positive neurons in the PVN. However, bilateral PVN microinjection of AAV-Ec-SOD decreased the number of NOX2-positive neurons and ASC-positive neurons in the PVN in the salt-induced hypertensive group (*p* < 0.05) (Figures 4A–C). Meanwhile, the double fluorescence staining results also indicated that NOX2 and ASC mainly expressed in the same cells. So we guessed that there probably exists an interaction between each other in the PVN.

### Effect of AAV-Ec-SOD on the Inflammation-Related Proteins in the PVN

**FIGURE 5:**
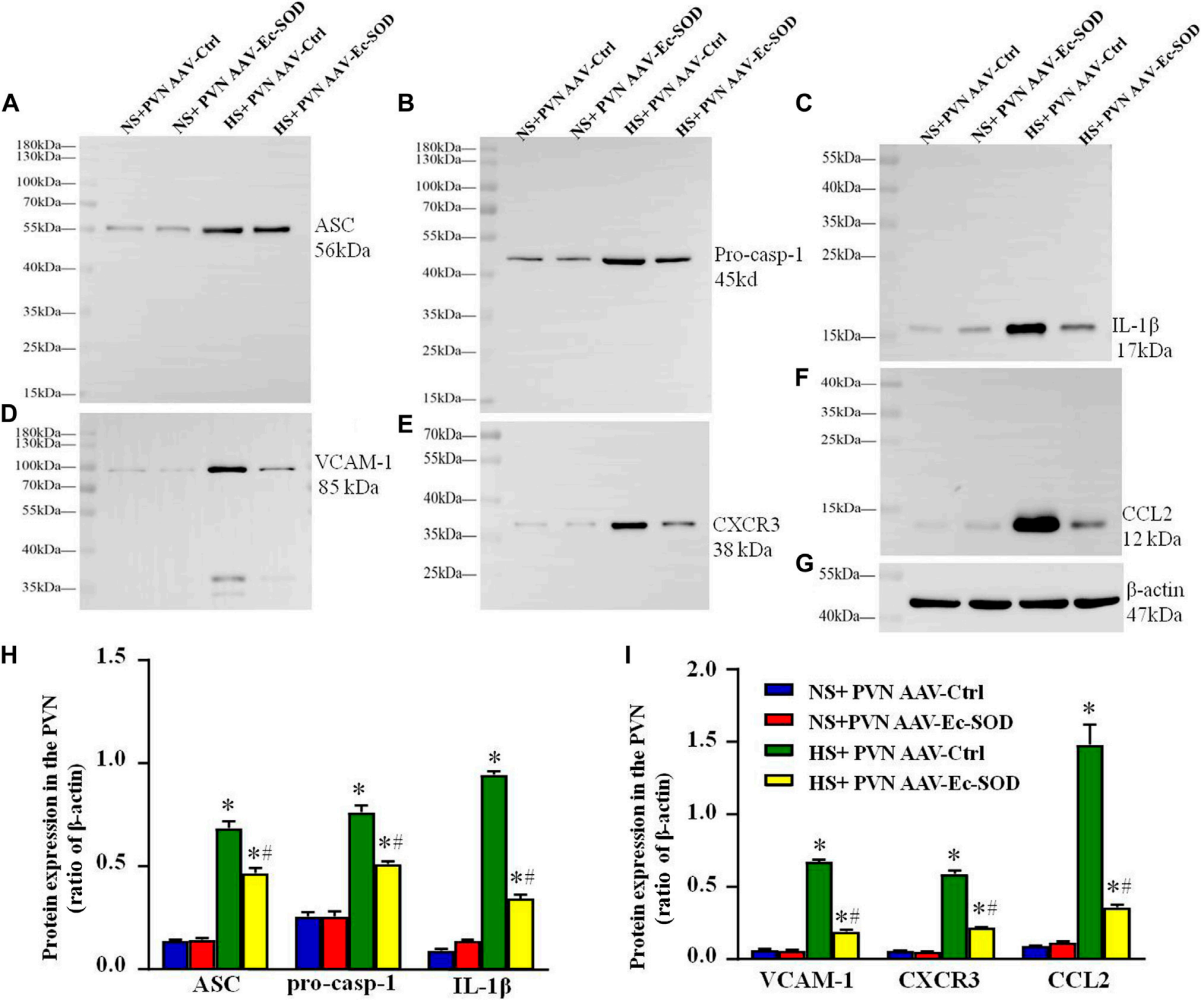
Effect of AAV-Ec-SOD on the inflammation proteins in the PVN. **(A–G)** Representative immunoblot and **(H–I)** densitometric analysis of PVN expression levels of ASC, pro-casp-1, IL-1β, VCAM-1, CXCR3, CCL2, and β-actin in different groups. Values are expressed as mean ± SEM (n = 4). *p < 0.05 NS + PVN AAV-Ctrl *vs.* HS + PVN AAV-Ec-SOD; ^***#***^ *p <* 0.05 HS + PVN AAV-Ctrl *vs.* HS + PVN AAV-Ec-SOD.

Western blotting results showed that inflammation-related proteins including ASC, VCAM-1, pro-casp-1, CXCR3, IL-1β, and CCL2 expression in the PVN were higher in the salt diet hypertensive groups than in the normal salt group (*p* < 0.05). Overexpression of Ec-SOD in the PVN significantly decreased the expression of ASC, VCAM-1, pro-casp-1, CXCR3, IL-1β, and CCL2 in the salt-induced hypertensive group (*p* < 0.05) (Figures 5A–I).

### Effect of AAV-Ec-SOD on the NOX2 and TH-Positive Neurons in the PVN

**FIGURE 6:**
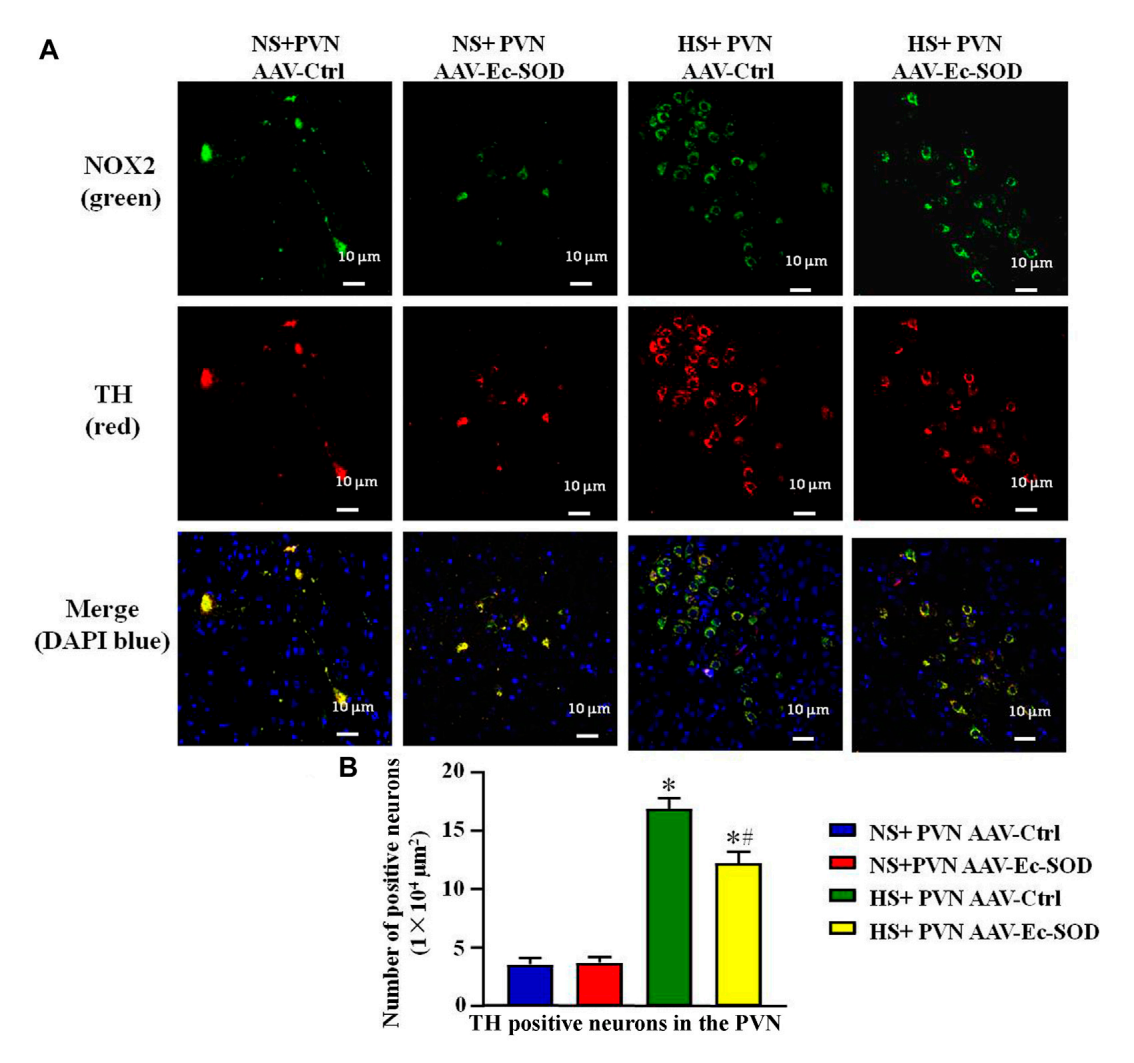
Effect of AAV-Ec-SOD on the NOX2 and TH-positive neurons in the PVN. **(A)** Double fluorescence staining for NOX2 and TH-positive neurons in the PVN in different groups. **(B)** Column diagram showing the TH-positive neurons in the PVN in different groups. Values are expressed as mean ± SEM (n = 6). *p < 0.05 NS + PVN AAV-Ctrl *vs.* HS + PVN AAV-Ec-SOD; ^***#***^ *p* < 0.05 HS + PVN AAV-Ctrl *vs.* HS + PVN AAV-Ec-SOD.

We measured PVN-positive neurons of NOX2 and TH by double fluorescence staining. The results showed that high-salt diet intake enhanced the number of NOX2-positive neurons and TH-positive neurons in the PVN. However, bilateral PVN microinjection of AAV-Ec-SOD decreased the number of NOX2-positive neurons and TH-positive neurons in the PVN in the excessive salt-induced hypertensive group (*p* < 0.05) (Figures 6A,B). It also similarly showed that NOX2 and TH mainly expressed in the same cell and probably have an effect on each other.

### Effect of AAV-Ec-SOD on the Neurotransmitter in the PVN

**FIGURE 7:**
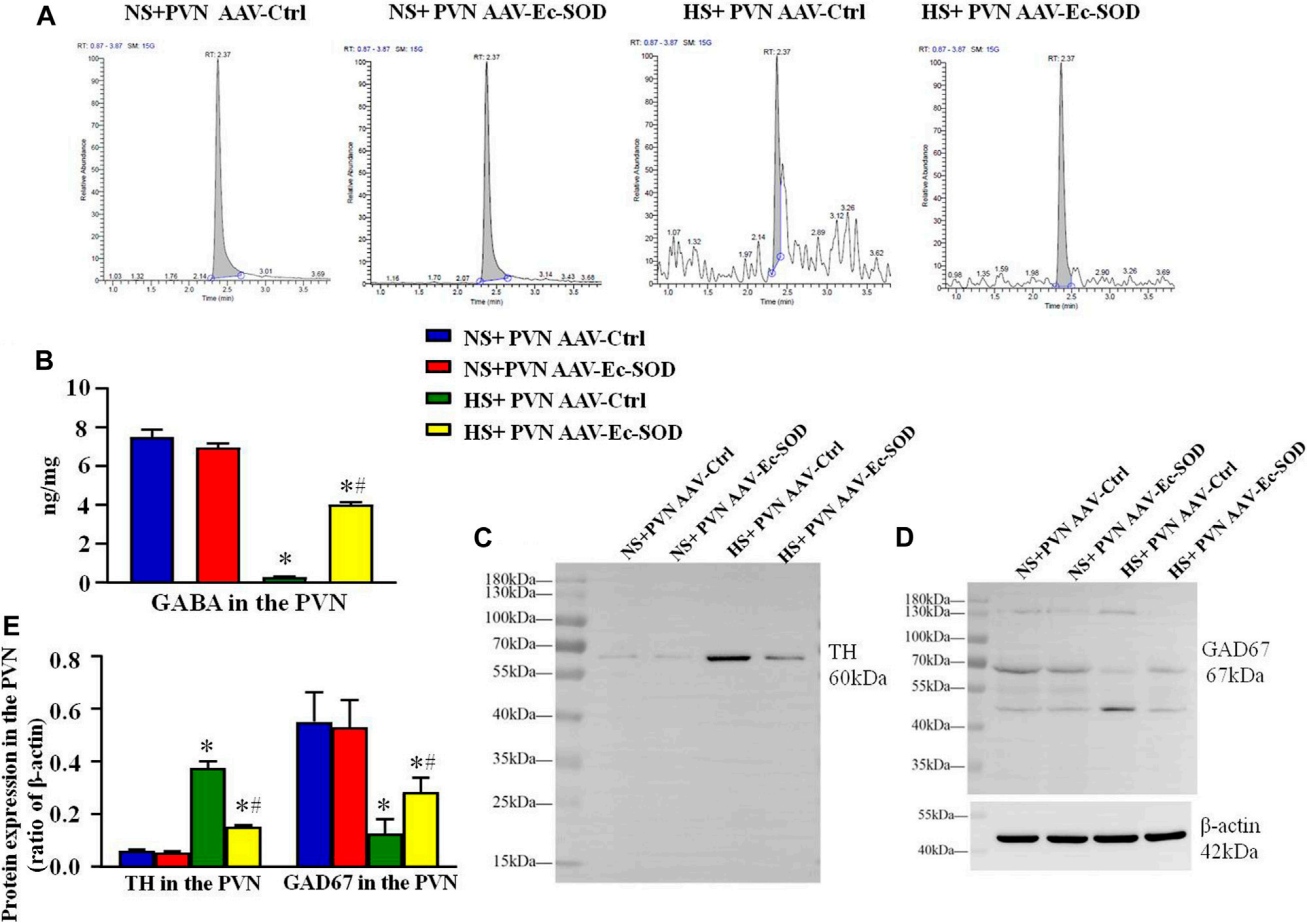
Effect of AAV-Ec-SOD on the neurotransmitter-related proteins in the PVN **(A)** High-performance liquid chromatography chromatograms of GABA in the PVN in different groups. **(B)** Column diagram for the PVN level of GABA in different groups. **(C–D)** Representative immunoblot and **(E)** densitometric analysis of PVN expression levels of TH and GAD67 in the different groups. Values are expressed as mean ± SEM (n = 4–6). *p < 0.05 NS + PVN AAV-Ctrl *vs.* HS + PVN AAV-Ec-SOD; ^***#***^ *p <* 0.05 HS + PVN AAV-Ctrl *vs.* HS + PVN AAV-Ec-SOD.

We measured the GABA in the PVN by HPLC-EC. It showed that excessive salt intake suppressed the GABA release in the PVN. However, overexpression of Ec-SOD in the PVN increased the level of GABA in the hypertensive group (*p* < 0.05) (Figures 7A,B).

Western blotting results showed that the TH expression in the PVN was higher in the excessive salt group than in the normal salt group (*p* < 0.05). Overexpression of Ec-SOD in the PVN significantly decreased the TH expression in the PVN in the hypertensive group (*p* < 0.05). As for the GAD67 expression in the PVN, excessive salt intake decreased the PVN level of GAD67, but bilateral PVN injection of AAV-Ec-SOD significantly elevated the GAD67 expression in the PVN in salt-induced hypertensive group (*p* < 0.05) (Figures 7C–E).

## Discussion

Long-term high salt augmented the NADP(H) oxidase-dependent ROS in the PVN and increased the blood pressure. Meanwhile, excessive sodium intake elevated the NLRP3-dependent inflammatory and broke the balance of neurotransmitters in the PVN. Ec-SOD, as an essential anti-oxidative enzyme, eliminated oxygen free radicals and decreased the blood pressure, probably through inhibition of the NLRP3-dependent inflammation and reduction of the excitatory neurotransmitter release in the PVN in salt-induced hypertension.

High salt intake not only induces oxidative stress in the peripheral tissues but also elevates the overproduction of ROS in the central system. There are numerous studies showing that excessive salt intake increases the level of sodium in the cerebrospinal fluid, which disturbs the Na^+^/K^+^ exchange mechanism on the cell membrane, and probably activates the downstream ROS signaling pathway (Nishimura et al., 1998; Wang and Leenen, 2002; Wang et al., 2003). In addition, Blaustein reviewed that elevated CSF [Na^+^] increases central sympathoexcitatory pathways, which is probably through the endogenous ouabain (endogenous digitalis-like factor)–ANG II neuromodulatory pathway in the central system (Blaustein et al., 2012). Our previous studies have proved that activated renin–angiotensin system (RAS) triggers the overproduction of ROS and increases the NAD(P)H oxidase activity and its subunits (NOX2 and NOX4) in the PVN, which produced the excessive ROS during the development of hypertension (Su et al., 2017). However, as for physiological responses, the essential role of antioxidant such as the SOD is to eliminate the excessive ROS so that it can protect the target cell and DNA from the ROS damage. As for the Ec-SOD, it is a unique SOD that exists in the extracellular matrix and cell surfaces that is primarily a protective barrier for the cell. There are two regions that expressed prominently Ec-SOD including tuberal hypothalamus and the blood–brain-barrier absence (Oury et al., 1999). This study also confirmed that Ec-SOD expressed in the PVN, which enhanced antioxidant capacity, reduced the ROS production, and finally contributed to the blood pressure decrease.

In this study, after injection of overexpression Ec-SOD, we also found that the expression of NLRP3 in the PVN is lower than in the hypertensive group. NLRP3 is one of (LRR)-containing (NLR) family members and is also the classic pathway that activates the inflammatory responses and inflammasome. There are many pathways or molecules such as TLR4/NF-κB pathway, ATP, poretoxin, and mitofusin2 (Mfn2), which can trigger the NLRP3-dependent inflammation (He et al., 2016). Activated NLRP3 oligomerizes the adaptor apoptosis-associated speck-like protein containing a CARD (ASC) to form ASC speck, which recruits pro-caspase-1 to mature caspase-1. And then the caspase-1 mediated the pro-IL-1β to the mature IL-1β so as to induce the inflammatory responses. Martinon (2010) reviewed that NAD(P)H oxidases (NOX) plays an important role in the activation of NLRP3 (Martinon, 2010). NOX is a family that includes NOX1, NOX2, NOX4, NOX5, and so on. However, the NOX2 regulates the ATP-mediated ROS production to activate NLRP3/NALP3. Therefore, the present study showed that excessive sodium intake increased the expression of NOX2 and NOX4 in the PVN while the expressions of NLRP3, ASC, pro-cas-1, and IL-1β are elevated. After inhibition of ROS by Ec-SOD antioxidant, the low activity of NAD(P)H oxidase in the PVN suppressed the NLRP3 and NLRP3-dependent downstream inflammatory responses, which indicated that oxidative stress activated the NLRP3-dependent inflammation *via* the NAD(P)H-oxidase components.

In addition, the PVN of the hypothalamus is reciprocally connected to other areas of the central nervous system that are involved in cardiovascular function (Swanson and Sawchenko, 1983) and is a significant central site for integration of sympathetic nerve activity (Swanson and Sawchenko, 1980) so as to adjust the cardiovascular responses. There are numerous evidences showing that PVN exists in the presympathetic neurons and delivers the signals to the downstream sympathetic nerve. Basgut et al. (2017) suggested that high salt diet on age-related hypertension increased NADPH oxidase activity in the hypothalamus and elevated the mRNA level of tyrosine hydroxylase (TH), an indicator for sympathoexcitation (Basgut et al., 2017). C-fos is currently used as a marker of neuronal activity/excitability and is closely associated with neural and behavioral responses to stimulation like inflammation, renin–angiotensin system (RAS) activation, injuries, infectious and some chemical stimulation, and so on1 (Krukoff et al., 1994; Herdegen et al., 1995; Lin et al., 1999; Remick et al., 2000; Lin and Wan, 2008; Ye et al., 2020). In this study, long-term salt diet evoked the oxidative stress through the overproduction of ROS, which activated the neurons evidenced by the increased number of c-fos-positive neurons in the PVN. Then, the excitability neurons induced the imbalance between excitatory (including NE and epinephrine, Glu) and inhibitory neurotransmitters such as GABA. Meanwhile, those excitatory neurotransmitters in the PVN, through the sympathetic nerve fibers, were transmitted to either RVLM (rostral ventrolateral medulla) then to IML (intermediolateral cell column) or to IML to augment the sympathetic nerve activity and elevate the blood pressure. It has been reported that bilateral PVN infusion of antioxidant decreased the TH expression and the plasma level of NE (Yi et al., 2016). In this study, we also observed that Ec-SOD decreased the TH expression but increased the GABA level and GAD67 expression in the PVN, which demonstrated the inhibition of oxidative stress by Ec-SOD restoring the imbalance of neurotransmitters.

However, the exact signaling pathway of the ROS acting with the neurotransmitters in the PVN is still unclear. Some researches pointed out that excessive superoxide anion (O_2_·−) can combine with nitric oxide (NO) to form the peroxynitrite anion (ONOO−), which has an irreversible effect on their transportation and physiological functions (Picon-Pages et al., 2019). However, the decreased nitric oxide synthase (NOS) activity leads to the neurotransmitter change. This may be the connecting point for the oxidative stress and neurotransmitters. We are also interested in exploring the related signaling pathway in the future.

**FIGURE 8:**
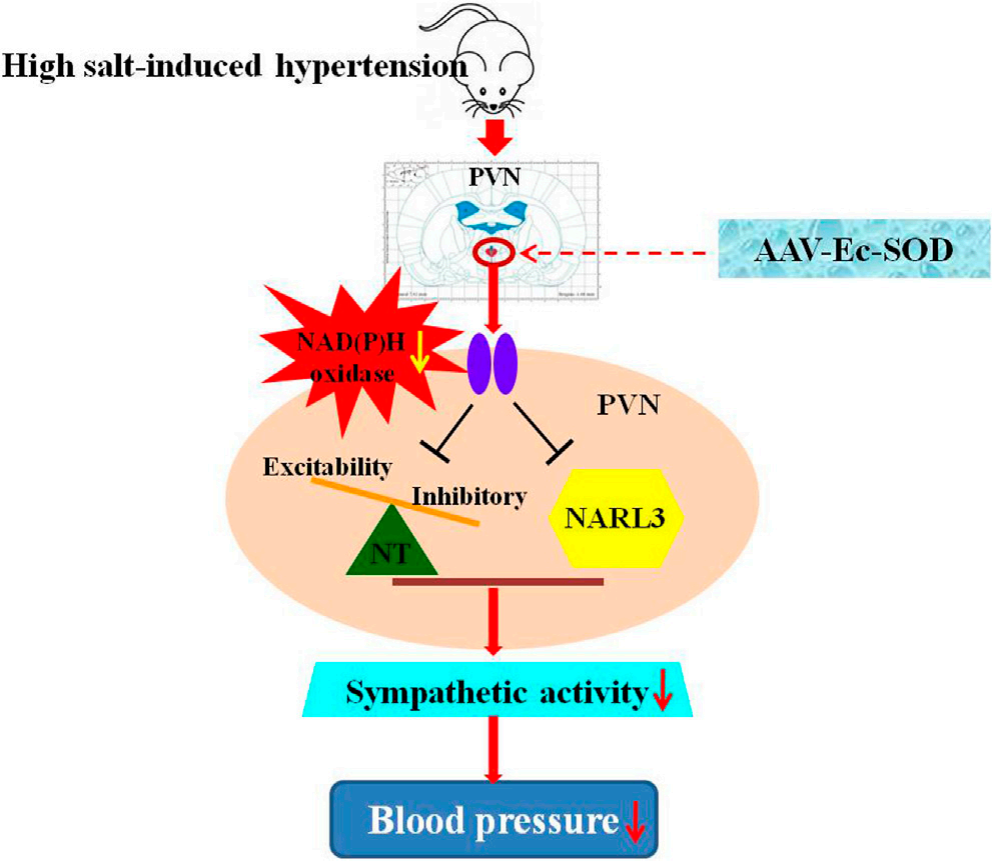
Schematic presentation of the proposed mechanisms of Ec-SOD in salt-induced hypertension. High salt diet increased the blood pressure and sympathetic activity, which contributed to hypertension. Bilateral PVN microinjection of the overexpression of AAV-Ec-SOD inhibits the oxidative stress in the PVN and decreases the blood pressure, probably through reducing the NLRP3-dependent inflammatory responses and restoring the imbalance of the neurotransmitters in the PVN during salt-induced hypertension.

In conclusion, high salt intake augments NAD(P)H oxidase–dependent ROS in the PVN, activates the NLRP3-dependent inflammatory reaction, and increases excitatory but suppresses inhibitory neurotransmitters in the PVN. The overexpression of Ec-SOD in the PVN has a positive effect on the decrease of sympathetic activity and blood pressure probably through inhibition of the NLRP3-dependent inflammation and elevation of the inhibitory neurotransmitter release in the PVN in salt-induced hypertension (Figure 8).

## Data Availability

The raw data supporting the conclusions of this article will be made available by the authors, without undue reservation.

## Supplementary Material

Supplement Figure S1 AAV-Ec-SOD vector construction and paraventricular nucleus Ec-SOD overexpression verification. **(A)** A structure of pHBAAV-CMV-MCS-3FLAG-T2A-ZsGreen AAV vector, which carries Ec-SOD and an empty construct or a ZsGreen gene as an indicator. **(B)** Graphic illustration of the AAV bilateral injection sites in the paraventricular nucleus of the rat brain. Arrows indicate the bilateral injection sites in the paraventricular nucleus region. Distribution of AAV-mediated ZsGreen expression in the PVN. 3v represents the third ventricle in brain. **(C,D)** Western blotting analysis and column diagram of Ec-SOD protein levels in the PVN of vehicle Ctrl, AAV-Ctrl, and AAV-Ec-SOD rat. **(E)** qPCR analysis and column diagram of Ec-SOD protein levels in the PVN of vehicle Ctrl, AAV-Ctrl, and AAV-Ec-SOD rat. Values are expressed as mean ± SEM (n=3–5). **p* < 0.05 *vs.* Ctrl or AAV-Ctrl.

## References

[B1] Alsaadi M. Tezcan G. Garanina E. E. Hamza S. McIntyre A. Rizvanov A. A. (2021). Doxycycline Attenuates Cancer Cell Growth by Suppressing NLRP3-Mediated Inflammation. Pharmaceuticals (Basel) 14 (9), 1. 10.3390/ph14090852 PMC8466018 34577552

[B2] Bai J. Yu X. J. Liu K. L. Wang F. F. Jing G. X. Li H. B. (2017a). Central Administration of Tert-Butylhydroquinone Attenuates Hypertension via Regulating Nrf2 Signaling in the Hypothalamic Paraventricular Nucleus of Hypertensive Rats. Toxicol. Appl. Pharmacol. 333, 100–109. 10.1016/j.taap.2017.08.012 28842207

[B3] Bai J. Yu X. J. Liu K. L. Wang F. F. Li H. B. Shi X. L. (2017b). Tert-butylhydroquinone Attenuates Oxidative Stress and Inflammation in Hypothalamic Paraventricular Nucleus in High Salt-Induced Hypertension. Toxicol. Lett. 281, 1–9. 10.1016/j.toxlet.2017.08.018 28844481

[B4] Basgut B. Whidden M. A. Kirichenko N. Woods M. Erdos B. Scarpace P. J. (2017). Effect of High-Salt Diet on Age-Related High Blood Pressure and Hypothalamic Redox Signaling. Pharmacology 100 (3-4), 105–114. 10.1159/000472259 28521325

[B5] Blaustein M. P. Leenen F. H. Chen L. Golovina V. A. Hamlyn J. M. Pallone T. L. (2012). How NaCl Raises Blood Pressure: a New Paradigm for the Pathogenesis of Salt-dependent Hypertension. Am. J. Physiol. Heart Circ. Physiol. 302 (5), H1031–H1049. 10.1152/ajpheart.00899.2011 22058154 PMC3311458

[B6] Boegehold M. A. Drenjancevic I. Lombard J. H. (2015). Salt, Angiotensin II, Superoxide, and Endothelial Function. Compr. Physiol. 6 (1), 215–254. 10.1002/cphy.c150008 26756632

[B7] Boegehold M. A. (2013). The Effect of High Salt Intake on Endothelial Function: Reduced Vascular Nitric Oxide in the Absence of Hypertension. J. Vasc. Res. 50 (6), 458–467. 10.1159/000355270 24192502

[B8] Channon K. M. (2004). Tetrahydrobiopterin: Regulator of Endothelial Nitric Oxide Synthase in Vascular Disease. Trends Cardiovasc. Med. 14 (8), 323–327. 10.1016/j.tcm.2004.10.003 15596110

[B9] Elks C. M. Reed S. D. Mariappan N. Shukitt-Hale B. Joseph J. A. Ingram D. K. (2011). A Blueberry-Enriched Diet Attenuates Nephropathy in a Rat Model of Hypertension via Reduction in Oxidative Stress. PLoS One 6 (9), e24028. 10.1371/journal.pone.0024028 21949690 PMC3174132

[B10] Fujita M. Ando K. Kawarazaki H. Kawarasaki C. Muraoka K. Ohtsu H. (2012). Sympathoexcitation by Brain Oxidative Stress Mediates Arterial Pressure Elevation in Salt-Induced Chronic Kidney Disease. Hypertension 59 (1), 105–112. 10.1161/HYPERTENSIONAHA.111.182923 22083162

[B11] Fujita M. Ando K. Nagae A. Fujita T. (2007). Sympathoexcitation by Oxidative Stress in the Brain Mediates Arterial Pressure Elevation in Salt-Sensitive Hypertension. Hypertension 50 (2), 360–367. 10.1161/HYPERTENSIONAHA.107.091009 17576857

[B13] Gu J. W. Bailey A. P. Tan W. Shparago M. Young E. (2008). Long-Term High Salt Diet Causes Hypertension and Decreases Renal Expression of Vascular Endothelial Growth Factor in Sprague-Dawley Rats. J. Am. Soc. Hypertens. 2 (4), 275–285. 10.1016/j.jash.2008.03.001 19122855 PMC2598434

[B12] Gu J. W. Manning R. D. Jr. Young E. Shparago M. Sartin B. Bailey A. P. (2009). Vascular Endothelial Growth Factor Receptor Inhibitor Enhances Dietary Salt-Induced Hypertension in Sprague-Dawley Rats. Am. J. Physiol. Regul. Integr. Comp. Physiol. 297 (1), R142–R148. 10.1152/ajpregu.90972.2008 19420288 PMC2711701

[B14] He Y. Hara H. Núñez G. (2016). Mechanism and Regulation of NLRP3 Inflammasome Activation. Trends Biochem. Sci. 41 (12), 1012–1021. 10.1016/j.tibs.2016.09.002 27669650 PMC5123939

[B15] He Y. Pan S. Xu M. He R. Huang W. Song P. (2017). Adeno-associated Virus 9-mediated Cdk5 Inhibitory Peptide Reverses Pathologic Changes and Behavioral Deficits in the Alzheimer's Disease Mouse Model. FASEB J. 31 (8), 3383–3392. 10.1096/fj.201700064R 28420695

[B16] Herdegen T. Kovary K. Buhl A. Bravo R. Zimmermann M. Gass P. (1995). Basal Expression of the Inducible Transcription Factors C-Jun, JunB, JunD, C-Fos, FosB, and Krox-24 in the Adult Rat Brain. J. Comp. Neurol. 354 (1), 39–56. 10.1002/cne.903540105 7615874

[B17] Huang B. S. Leenen F. H. (1999). Brain Renin-Angiotensin System and Ouabain-Induced Sympathetic Hyperactivity and Hypertension in Wistar Rats. Hypertension 34 (1), 107–112. 10.1161/01.hyp.34.1.107 10406832

[B18] Huang B. S. Leenen F. H. (1996). Sympathoexcitatory and Pressor Responses to Increased Brain Sodium and Ouabain Are Mediated via Brain ANG II. Am. J. Physiol. 270 (1 Pt 2), H275–H280. 10.1152/ajpheart.1996.270.1.H275 8769762

[B19] Huo C. J. Yu X. J. Sun Y. J. Li H. B. Su Q. Bai J. (2020). Irisin Lowers Blood Pressure by Activating the Nrf2 Signaling Pathway in the Hypothalamic Paraventricular Nucleus of Spontaneously Hypertensive Rats. Toxicol. Appl. Pharmacol. 394, 114953. 10.1016/j.taap.2020.114953 32165127

[B20] Jo E. K. Kim J. K. Shin D. M. Sasakawa C. (2016). Molecular Mechanisms Regulating NLRP3 Inflammasome Activation. Cell Mol Immunol 13 (2), 148–159. 10.1038/cmi.2015.95 26549800 PMC4786634

[B21] Kido M. Ando K. Onozato M. L. Tojo A. Yoshikawa M. Ogita T. (2008). Protective Effect of Dietary Potassium against Vascular Injury in Salt-Sensitive Hypertension. Hypertension 51 (2), 225–231. 10.1161/HYPERTENSIONAHA.107.098251 18158352

[B22] Krukoff T. L. Harris K. H. Linetsky E. Jhamandas J. H. (1994). Expression of C-Fos Protein in Rat Brain Elicited by Electrical and Chemical Stimulation of the Hypothalamic Paraventricular Nucleus. Neuroendocrinology 59 (6), 590–602. 10.1159/000126709 7916128

[B23] Li H. B. Li X. Huo C. J. Su Q. Guo J. Yuan Z. Y. (2016). TLR4/MyD88/NF-κB Signaling and PPAR-γ within the Paraventricular Nucleus Are Involved in the Effects of Telmisartan in Hypertension. Toxicol. Appl. Pharmacol. 305, 93–102. 10.1016/j.taap.2016.06.014 27292124

[B24] Li H. B. Yang T. Richards E. M. Pepine C. J. Raizada M. K. (2020). Maternal Treatment with Captopril Persistently Alters Gut-Brain Communication and Attenuates Hypertension of Male Offspring. Hypertension 75 (5), 1315–1324. 10.1161/HYPERTENSIONAHA.120.14736 32200676 PMC7145738

[B25] Lin H. C. Wan F. J. (2008). Hyperbaric Oxygenation Reduces Overexpression of C-Fos and Oxidative Stress in the Brain Stem of Experimental Endotoxemic Rats. Intensive Care Med. 34 (6), 1122–1132. 10.1007/s00134-007-0986-3 18193191

[B26] Lin H. C. Wan F. J. Kang B. H. Wu C. C. Tseng C. J. (1999). Systemic Administration of Lipopolysaccharide Induces Release of Nitric Oxide and Glutamate and C-Fos Expression in the Nucleus Tractus Solitarii of Rats. Hypertension 33 (5), 1218–1224. 10.1161/01.hyp.33.5.1218 10334815

[B27] Lupia E. Spatola T. Cuccurullo A. Bosco O. Mariano F. Pucci A. (2010). Thrombopoietin Modulates Cardiac Contractility *In Vitro* and Contributes to Myocardial Depressing Activity of Septic Shock Serum. Basic Res. Cardiol. 105 (5), 609–620. 10.1007/s00395-010-0103-6 20467749

[B28] Marklund S. L. Holme E. Hellner L. (1982). Superoxide Dismutase in Extracellular Fluids. Clin. Chim. Acta 126 (1), 41–51. 10.1016/0009-8981(82)90360-6 7172448

[B29] Martinon F. (2010). Signaling by ROS Drives Inflammasome Activation. Eur. J. Immunol. 40 (3), 616–619. 10.1002/eji.200940168 20201014

[B30] Matsui H. Shimosawa T. Uetake Y. Wang H. Ogura S. Kaneko T. (2006). Protective Effect of Potassium against the Hypertensive Cardiac Dysfunction: Association with Reactive Oxygen Species Reduction. Hypertension 48 (2), 225–231. 10.1161/01.HYP.0000232617.48372.cb 16818802

[B31] McKie G. L. Medak K. D. Knuth C. M. Shamshoum H. Townsend L. K. Peppler W. T. (2019). Housing Temperature Affects the Acute and Chronic Metabolic Adaptations to Exercise in Mice. J. Physiol. 597 (17), 4581–4600. 10.1113/JP278221 31297830

[B32] MohanKumar S. M. MohanKumar P. S. Quadri S. K. (1998). Specificity of Interleukin-1beta-Induced Changes in Monoamine Concentrations in Hypothalamic Nuclei: Blockade by Interleukin-1 Receptor Antagonist. Brain Res. Bull. 47 (1), 29–34. 10.1016/s0361-9230(98)00037-9 9766386

[B33] Nishimura M. Ohtsuka K. Nanbu A. Takahashi H. Yoshimura M. (1998). Benzamil Blockade of Brain Na+ Channels Averts Na(+)-Induced Hypertension in Rats. Am. J. Physiol. 274 (3), R635–R644. 10.1152/ajpregu.1998.274.3.R635 9530228

[B34] Nurkiewicz T. R. Boegehold M. A. (2007). High Salt Intake Reduces Endothelium-dependent Dilation of Mouse Arterioles via Superoxide Anion Generated from Nitric Oxide Synthase. Am. J. Physiol. Regul. Integr. Comp. Physiol. 292 (4), R1550–R1556. 10.1152/ajpregu.00703.2006 17138723

[B35] Oury T. D. Card J. P. Klann E. (1999). Localization of Extracellular Superoxide Dismutase in Adult Mouse Brain. Brain Res. 850 (1-2), 96–103. 10.1016/s0006-8993(99)02103-4 10629753

[B36] Patel Y. Joseph J. (2020). Sodium Intake and Heart Failure. Int. J. Mol. Sci. 21 (24), 1. 10.3390/ijms21249474 PMC7763082 33322108

[B37] Picón-Pagès P. Garcia-Buendia J. Muñoz F. J. (2019). Functions and Dysfunctions of Nitric Oxide in Brain. Biochim. Biophys. Acta Mol. Basis Dis. 1865 (8), 1949–1967. 10.1016/j.bbadis.2018.11.007 30500433

[B38] Remick D. G. Newcomb D. E. Bolgos G. L. Call D. R. (2000). Comparison of the Mortality and Inflammatory Response of Two Models of Sepsis: Lipopolysaccharide vs. Cecal Ligation and Puncture. Shock 13 (2), 110–116. 10.1097/00024382-200013020-00004 10670840

[B39] Sasaki T. Abe Y. Takayama M. Adachi T. Okano H. Hirose N. (2021). Association Among Extracellular Superoxide Dismutase Genotype, Plasma Concentration, and Comorbidity in the Very Old and Centenarians. Sci. Rep. 11 (1), 8539. 10.1038/s41598-021-87982-6 33879836 PMC8058336

[B40] Schroder K. Tschopp J. (2010). The Inflammasomes. Cell 140 (6), 821–832. 10.1016/j.cell.2010.01.040 20303873

[B41] Selektor Y. Weber K. T. (2008). The Salt-Avid State of Congestive Heart Failure Revisited. Am. J. Med. Sci. 335 (3), 209–218. 10.1097/MAJ.0b013e3181591da0 18344695

[B42] Su Q. Huo C. J. Li H. B. Liu K. L. Li X. Yang Q. (2017). Renin-angiotensin System Acting on Reactive Oxygen Species in Paraventricular Nucleus Induces Sympathetic Activation via AT1R/PKCγ/Rac1 Pathway in Salt-Induced Hypertension. Sci. Rep. 7, 43107. 10.1038/srep43107 28338001 PMC5364504

[B43] Su Q. Liu J. J. Cui W. Shi X. L. Guo J. Li H. B. (2016). Alpha Lipoic Acid Supplementation Attenuates Reactive Oxygen Species in Hypothalamic Paraventricular Nucleus and Sympathoexcitation in High Salt-Induced Hypertension. Toxicol. Lett. 241, 152–158. 10.1016/j.toxlet.2015.10.019 26518973

[B44] Su Q. Qin D. N. Wang F. X. Ren J. Li H. B. Zhang M. (2014). Inhibition of Reactive Oxygen Species in Hypothalamic Paraventricular Nucleus Attenuates the Renin-Angiotensin System and Proinflammatory Cytokines in Hypertension. Toxicol. Appl. Pharmacol. 276 (2), 115–120. 10.1016/j.taap.2014.02.002 24576725

[B45] Swanson L. W. Sawchenko P. E. (1983). Hypothalamic Integration: Organization of the Paraventricular and Supraoptic Nuclei. Annu. Rev. Neurosci. 6, 269–324. 10.1146/annurev.ne.06.030183.001413 6132586

[B46] Swanson L. W. Sawchenko P. E. (1980). Paraventricular Nucleus: a Site for the Integration of Neuroendocrine and Autonomic Mechanisms. Neuroendocrinology 31 (6), 410–417. 10.1159/000123111 6109264

[B47] Thorsdottir D. Einwag Z. Erdos B. (2021). BDNF Shifts Excitatory-Inhibitory Balance in the Paraventricular Nucleus of the Hypothalamus to Elevate Blood Pressure. J. Neurophysiol. 126, 1209–1220. 10.1152/jn.00247.2021 34406887 PMC8560414

[B48] Wang H. Huang B. S. Leenen F. H. (2003). Brain Sodium Channels and Ouabainlike Compounds Mediate central Aldosterone-Induced Hypertension. Am. J. Physiol. Heart Circ. Physiol. 285 (6), H2516–H2523. 10.1152/ajpheart.00299.2003 12933342

[B49] Wang H. Leenen F. H. (2002). Brain Sodium Channels Mediate Increases in Brain "ouabain" and Blood Pressure in Dahl S Rats. Hypertension 40 (1), 96–100. 10.1161/01.hyp.0000022659.17774.e4 12105145

[B50] Wang M. L. Kang Y. M. Li X. G. Su Q. Li H. B. Liu K. L. (2018). Central Blockade of NLRP3 Reduces Blood Pressure via Regulating Inflammation Microenvironment and Neurohormonal Excitation in Salt-Induced Prehypertensive Rats. J. Neuroinflammation 15 (1), 95. 10.1186/s12974-018-1131-7 29573749 PMC5866519

[B51] Weinstock M. Gorodetsky E. Kalman R. (1996). Renal Denervation Prevents Sodium Retention and Hypertension in Salt-Sensitive Rabbits with Genetic Baroreflex Impairment. Clin. Sci. (Lond) 90 (4), 287–293. 10.1042/cs0900287 8777835

[B52] Xia W. J. Xu M. L. Yu X. J. Du M. M. Li X. H. Yang T. (2021). Antihypertensive Effects of Exercise Involve Reshaping of Gut Microbiota and Improvement of Gut-Brain axis in Spontaneously Hypertensive Rat. Gut Microbes 13 (1), 1–24. 10.1080/19490976.2020.1854642 PMC7781639 33382364

[B53] Xie L. Mao X. Jin K. Greenberg D. A. (2013). Vascular Endothelial Growth Factor-B Expression in Postischemic Rat Brain. Vasc. Cell 5, 8. 10.1186/2045-824X-5-8 23601533 PMC3671984

[B54] Ye C. Qiu Y. Zhang F. Chen A. D. Zhou H. Wang J. J. (2020). Chemical Stimulation of Renal Tissue Induces Sympathetic Activation and a Pressor Response via the Paraventricular Nucleus in Rats. Neurosci. Bull. 36 (2), 143–152. 10.1007/s12264-019-00417-1 31392556 PMC6977808

[B55] Yi Q. Y. Li H. B. Qi J. Yu X. J. Huo C. J. Li X. (2016). Chronic Infusion of Epigallocatechin-3-O-Gallate into the Hypothalamic Paraventricular Nucleus Attenuates Hypertension and Sympathoexcitation by Restoring Neurotransmitters and Cytokines. Toxicol. Lett. 262, 105–113. 10.1016/j.toxlet.2016.09.010 27659729

[B56] Yu X. J. Zhao Y. N. Hou Y. K. Li H. B. Xia W. J. Gao H. L. (2019). Chronic Intracerebroventricular Infusion of Metformin Inhibits Salt-Sensitive Hypertension via Attenuation of Oxidative Stress and Neurohormonal Excitation in Rat Paraventricular Nucleus. Neurosci. Bull. 35 (1), 57–66. 10.1007/s12264-018-0308-5 30426340 PMC6357266

[B57] Zheng L. Yu M. Lin R. Wang Y. Zhuo Z. Cheng N. (2020). Rhythmic Light Flicker Rescues Hippocampal Low Gamma and Protects Ischemic Neurons by Enhancing Presynaptic Plasticity. Nat. Commun. 11 (1), 3012. 10.1038/s41467-020-16826-0 32541656 PMC7296037

[B58] Zhou R. Yazdi A. S. Menu P. Tschopp J. (2011). A Role for Mitochondria in NLRP3 Inflammasome Activation. Nature 469 (7329), 221–225. 10.1038/nature09663 21124315

[B59] Zhu X. Li X. Zhu M. Xu K. Yang L. Han B. (2018). Metalloprotease Adam10 Suppresses Epilepsy through Repression of Hippocampal Neuroinflammation. J. Neuroinflammation 15 (1), 221. 10.1186/s12974-018-1260-z 30075790 PMC6091106

