# Supplementary figures and images for: Bilateral Paraventricular Nucleus Upregulation of Extracellular Superoxide Dismutase Decreases Blood Pressure by Regulation of the NLRP3 and Neurotransmitters in Salt-Induced Hypertensive Rats
Source: Front Pharmacol. 2021 Nov 25;12:756671. doi: 10.3389/fphar.2021.756671 (PMC8656229; doi:10.3389/fphar.2021.756671)

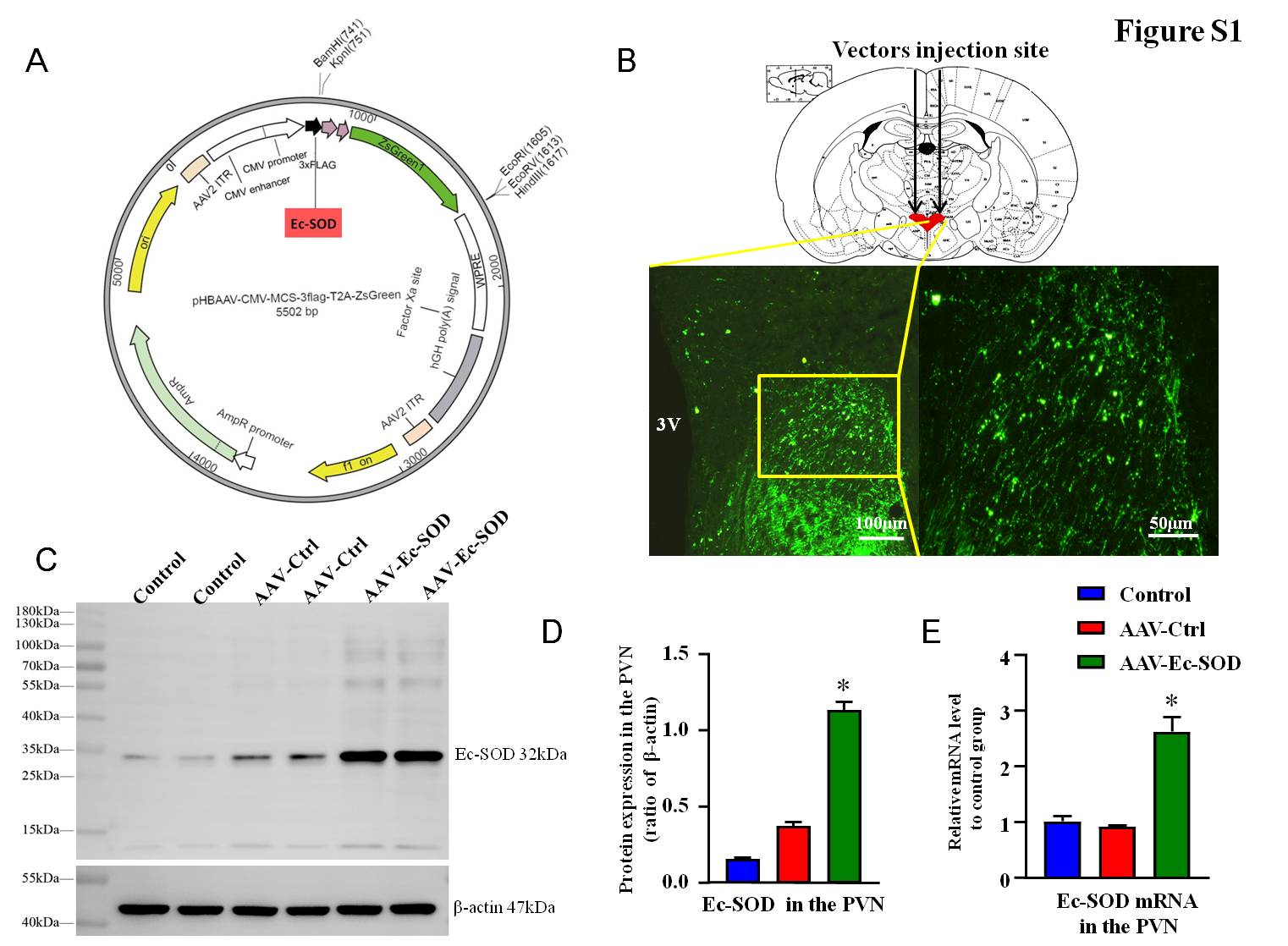

Supplement: Supplementary file 1 [file Image1.jpg]
